# Minimally invasive surgery for paravertebral or psoas abscess with spinal tuberculosis — a long-term retrospective study of 106 cases

**DOI:** 10.1186/s12891-020-03344-9

**Published:** 2020-06-06

**Authors:** Zhifa Zhang, Yongyu Hao, Xiangyu Wang, Zhirong Zheng, Xuelin Zhao, Chunguo Wang, Xifeng Zhang, Xuesong Zhang

**Affiliations:** Department of Orthopaedics, the PLA General Hospital, Beijing, 100000 China

**Keywords:** Minimally invasive surgery, Paravertebral abscess, Psoas abscess, Spinal tuberculosis

## Abstract

**Background:**

Minimally invasive surgery (MIS) is a common treatment option for paravertebral or psoas abscesses (PAs) in patients with spinal tuberculosis (ST). However, its efficacy remains controversial. The aim of the study was to evaluate the efficacy of MIS for PA with ST combined with anti-tuberculous chemotherapy.

**Methods:**

A total of 106 consecutive patients who underwent MIS for ST with PA from January 2002 to Oct 2012 were reviewed. The MIS involved computed tomography (CT)-guided percutaneous catheter drainage and percutaneous catheter infusion chemotherapy. Clinical outcomes were evaluated based on the changes observed on preoperative and postoperative physical examination, inflammatory marker testing, and magnetic resonance imaging (MRI).

**Results:**

The mean follow-up period was 7.21 ± 3.15 years. All surgeries were successfully completed under CT-guidance without intraoperative complications and all patients experienced immediate relief of their symptoms, which included fever and back pain. The preoperatively elevated erythrocyte sedimentation rate and C-reactive protein values returned to normal at a mean period of 3 months postoperatively. Solid bony union was observed in 106 patients and no abscesses were found on MRI examination.

**Conclusion:**

MIS carries advantages in terms of less invasiveness, precise drainage, and enhanced local drug concentration. While the technique has not been fully characterized and clinically prove, its use in addition to conservative chemotherapy and open debridement and instrumental fixation may be recommended for patients with ST and PA.

## Background

According to the World Health Organization’s (WHO) 2015 Global Tuberculosis (TB) Report, newly emerging multidrug-resistant TB and TB alongside HIV have become a leading cause of death worldwide [1]. It was reported that China possesses the third highest prevalence of TB worldwide. Tb, which is mentioned in the historical literature, is caused by *Mycobacterium tuberculosis*, remains an immense public health concern both in China and globally [2].

Spinal tuberculosis (ST), which was first described in the European population by Percival Pott in 1779 [3], is found in half of all cases of extrapulmonary TB, with a predilection for the thoracic and lumbar regions [4].

Psoas or paravertebral abscesses (PAs), which were first reported by Mynter in 1881 [5], is a common complication associated with ST [6]. Tuberculous spondylitis-induced PA is not a rare entity in China, especially in developing areas [7]. The objective of treatment for PA with thoracolumbar or lumbar TB is the complete drainage of abscess with regular anti-TB chemotherapy. We have employed a minimally invasive approach involving PA elimination by percutaneous catheter drainage (PCD) and intervertebral percutaneous catheter infusion (PCI) chemotherapy for treating the TB lesions. This study aimed to review the outcomes achieved by our minimally invasive approach to PA with TS.

## Methods

The study was approved by the ethics committee of the PLA General Hospital (Beijing, China) and carried out in accordance with the principles stated in the Declaration of Helsinki. In addition, written informed consent was obtained from all patients and their legal guardians to use and publish their medical images. No identifying information is shown in the images.

**Fig. 1:**
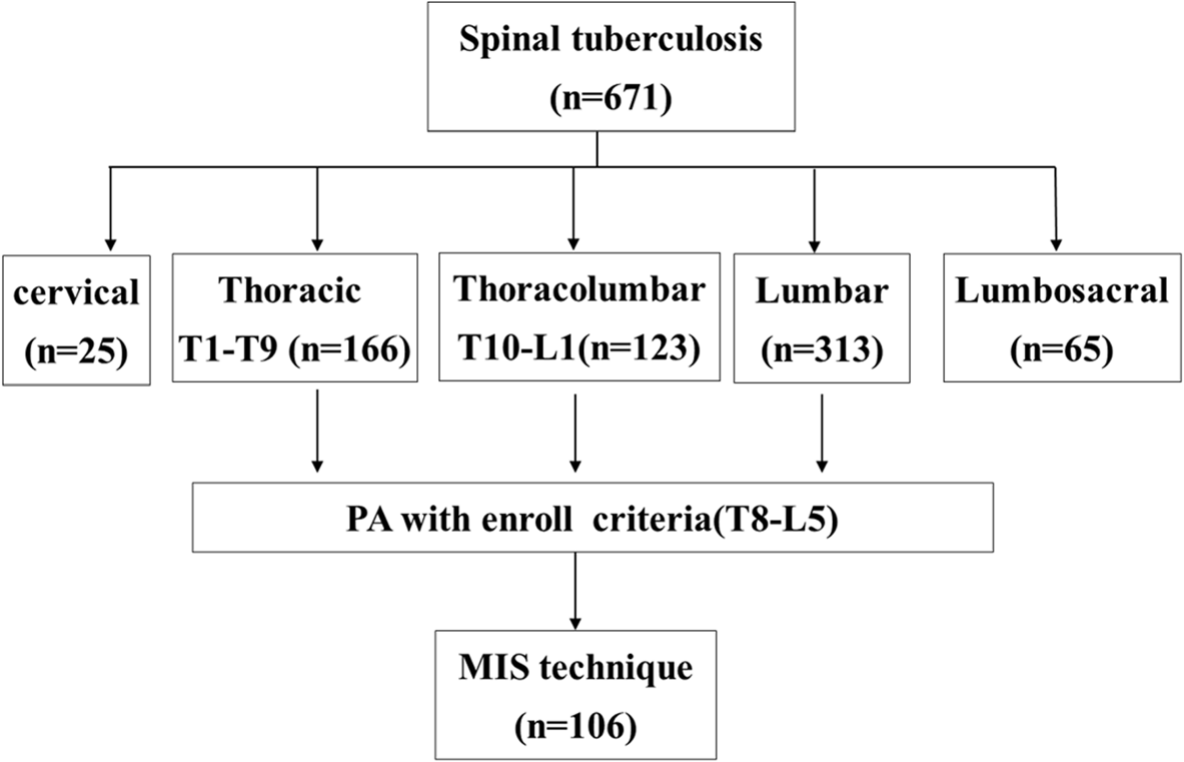
From January 2002 to October 2012, 671 consecutive spinal tuberculosis cases were retrospectively analyzed. 106 cases with PA treated by MIS technique were enrolled in the study

ST patients with PA treated at our center using our minimally invasive surgery (MIS) technique from January 2002 to October 2012 were retrospectively reviewed. The general inclusion criteria were significant cavity formation of paravertebral abscess or PA secondary to spinal TB involving the T8-L5 vertebrae without active pulmonary TB. Patients with any of the following exclusion criteria did not undergo MIS: (1) severe coagulation disorders; (2) concomitant obvious nerve or spinal cord compression symptoms with a Frankel classification level above grade C (not including grade C); (3) severe destruction of the vertebral body and obvious kyphosis or spinal instability requiring correction observed on the radiological examinations; (4) and co-infection with HIV/AIDS or other immune-compromising. Accordingly, 671 consecutive patients with ST were identified and 106 were included in this study (Fig. 1). The clinical data of the patients are shown in Table 1.

**Table 1:** Patient characteristics

Characteristic	No. (%)
**Involved vertebra**	
Thoracic vertebrae (T8–T10)	22 (15.4)
Thoracolumbar vertebrae (T11–L2)	63 (51.3)
Lumbar vertebrae (L3-L5)	21 (33.3)
**Abscess location**	
Paravertebral abscess	90 (51.3)
Psoas abscess	47 (41.0)
Epidural abscess	47 (41.0)
**Complications**	
Kyphosis	7 (35.9)
Recurrent abscess	4 (64.1)

### Diagnosis

The diagnosis of PA associated with tuberculous thoracolumbar or lumbar spondylitis was preoperatively based on the clinical symptoms, such as mild fever, night-sweats, back pain, and fatigue [8]; laboratory examinations such C-reactive protein (CRP), erythrocyte sedimentation rate (ESR), purified protein derivative, and the interferon gamma release assay [9];, and imaging examinations such as X-ray, computed tomography (CT), and magnetic resonance imaging (MRI) [6]. All patients were administered experimental anti-TB therapy for 2–4 weeks preoperatively [10]. The diagnoses of tuberculosis-induced PA was confirmed by pathological examination, acid-fast staining and/or culture of mycobacterium TB [2].

### MIS technique

All operations were performed under local anesthesia. PCI chemotherapy infusion via an epidural tube was directed towards the intervertebral TB lesions and PCD via a double-lumen was performed for drainage of the abscess cavity based on preoperative CT and MRI findings and intraoperative CT guidance. Disposable AS-E/S II anesthesia puncture kits were provided by Shandong Weigao Group Medical Polymer Co., Ltd. (Weihai, P. R. China).

**Fig. 2:**
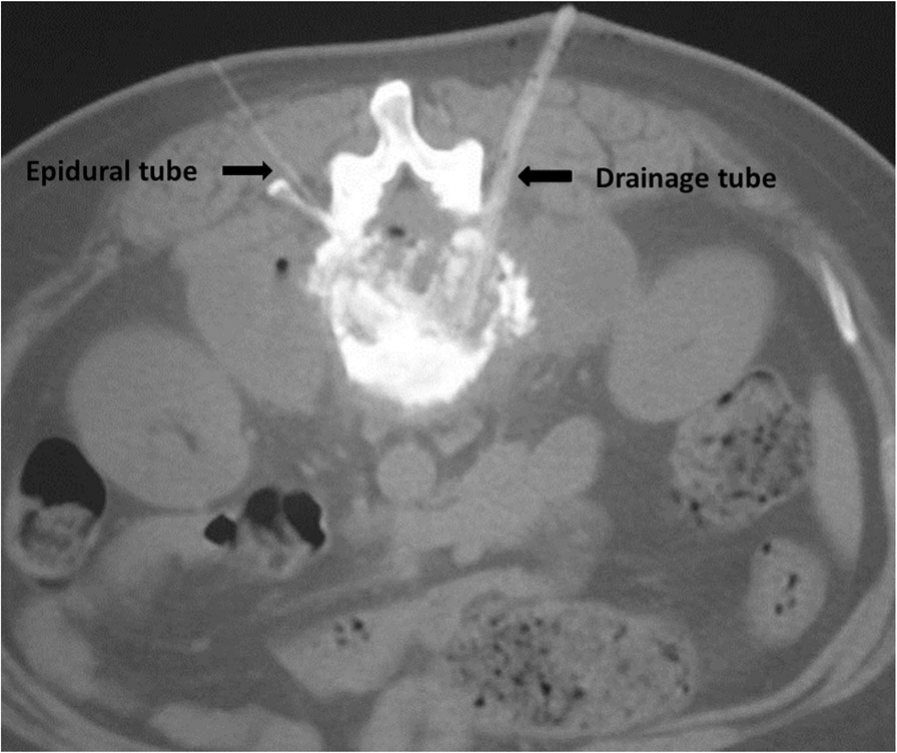
For thoracic vertebrae lesions, placement of epidural tubes were performed above the transverse process. For lumbar vertebrae, placement of abscess drainage tubes were performed from the Kambin triangle into the intervertebral space

For thoracic vertebral lesions, the epidural tubes were placed above the transverse process and into the intervertebral space, whereas for lumbar lesions they were placed through the Kambin triangle into the intervertebral space. Considering the need for an adequate volume to cover vertebral infectious lesions, double PCI chemotherapy was administered from both sides via 2 epidural tubes to increase drug concentration. For psoas abscesses or PAs, placement of the double-lumen was performed vertically from the dorsal side into abscess cavity (Fig. 2).

The details of the surgical procedures are as follows. For PCI chemotherapy, once CT scanning confirmed placement of the epidural needle in the lesion’s center, the inner core was removed and the epidural tube placed. When the final position of the epidural tube was reconfirmed, the needle was removed and the tube was attached tightly to the skin with medical paste. When needed, the same procedure would be performed on the other side.

**Fig. 3:**
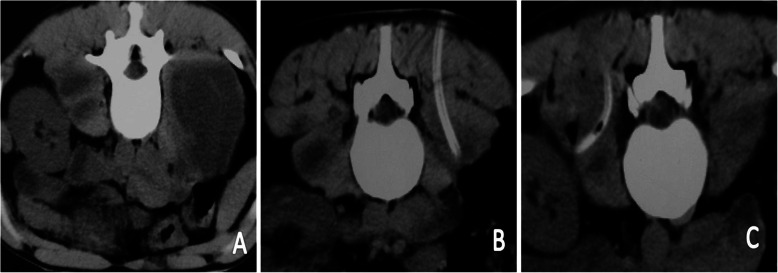
(**a**) The bilateral psoas abscess cavities were observed in the process of percutaneous puncture. (**b**) and (**c**) The abscess drainage tubes were placed in the middle of the abscess cavities under CT-guidance

For PCD via a double-lumen tube, once puncture had been successfully accomplished, the inner core was replaced by a guidewire. Using the guidewire, a 5-mm OD expansion pipe with a tapered front was placed inside. A CT scan was performed to confirm proper placement of the 5-mm OD working tube. The double-lumen tube was them placed through the working tube in the PA cavity and CT confirmation was repeated, followed by careful removal of the working tube (Fig. 3).

### Postoperative continuous drainage and local infusion chemotherapy

After the catheterization process was completed, the infusion tube was sealed with heparin saline and the drainage tube was connected to a negative-pressure sterile drainage bag. The abscess material obtained from the pretreatment puncture was subjected to TB and standard bacterial culture along with drug susceptibility testing [11, 12].

During local infusion of chemotherapy and abscess drainage, 24-h continuous lavage and drainage was performed using 500 ml of saline + 1 g isoniazid via the PCD tube over a period of 3–6 weeks. Chemotherapy (0.2 g isoniazid) was infused via the PCI tube over a period of 1 month [13]. If necessary, local chemotherapy infusion was extended to 3 months [14]. Extubation was not performed until the fluid drained from the abscess cavity was clear and without obvious necrotic tissue [15, 16]. After removal of the infusion and drainage tubes and patient discharge, oral anti-TB drugs were continued for 1 year, while functional rehabilitation and lumbar back muscle exercises were simultaneously performed under the protection of a thoracolumbar brace [17].

### Efficacy evaluation

**Fig. 4:**
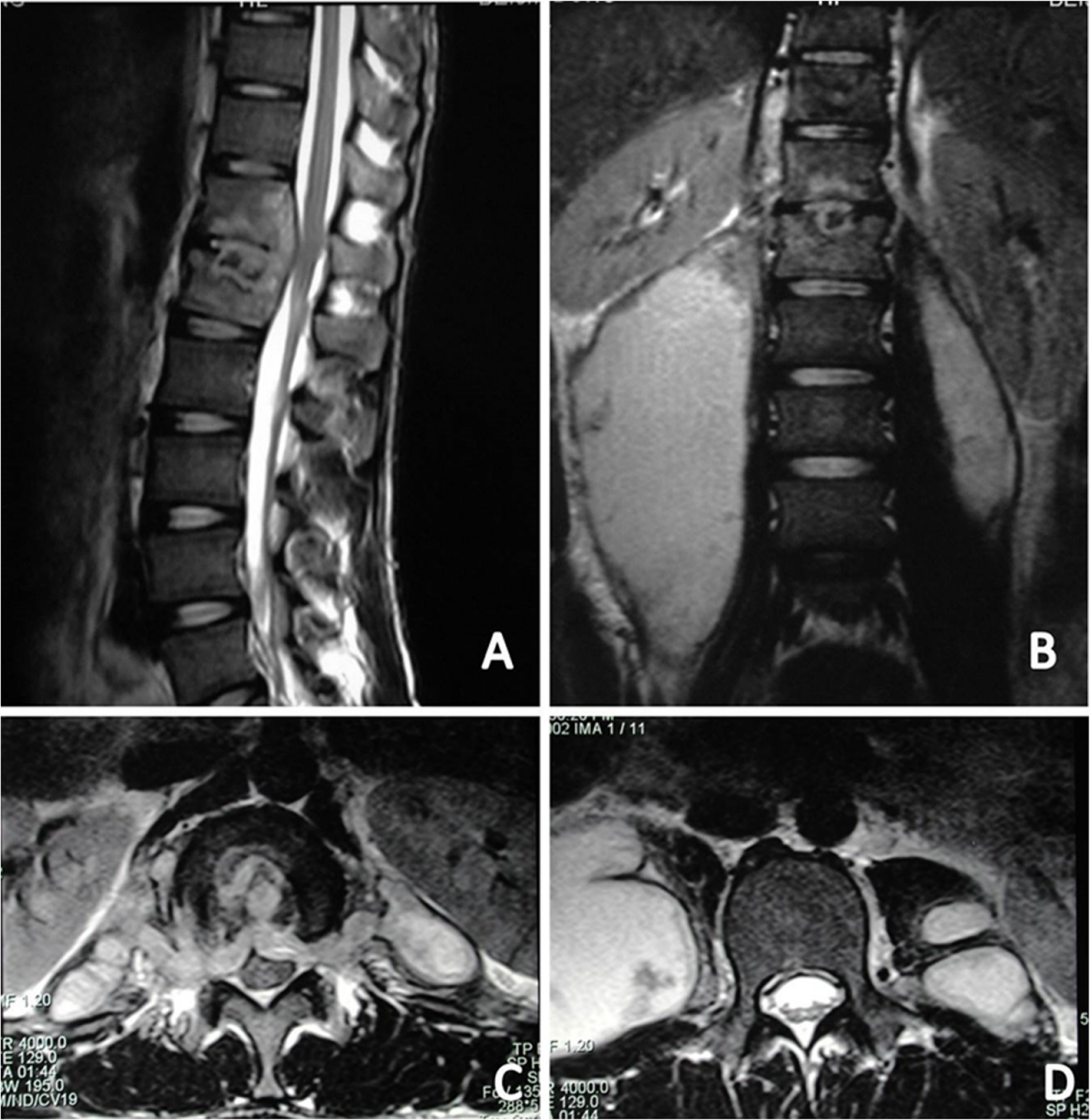
MRI showed that both sides of psoas abscess existed in a 22-year-old male patient with tuberculous spondylitis of T12-L1. **a** Sagittal T2-weighted MRI demonstrated a T12-L1 infection source and associated bilateral psoas muscle abscess. **b** Coronal T2-weighted magnetic resonance imaging (MRI) demonstrated that the right kidney was not in the right place because of the right huge psoas abscess. **c** and **d** showed the para-vertebral abscess and psoas abscess existed in the relative section of the axial section

**Fig. 5:**
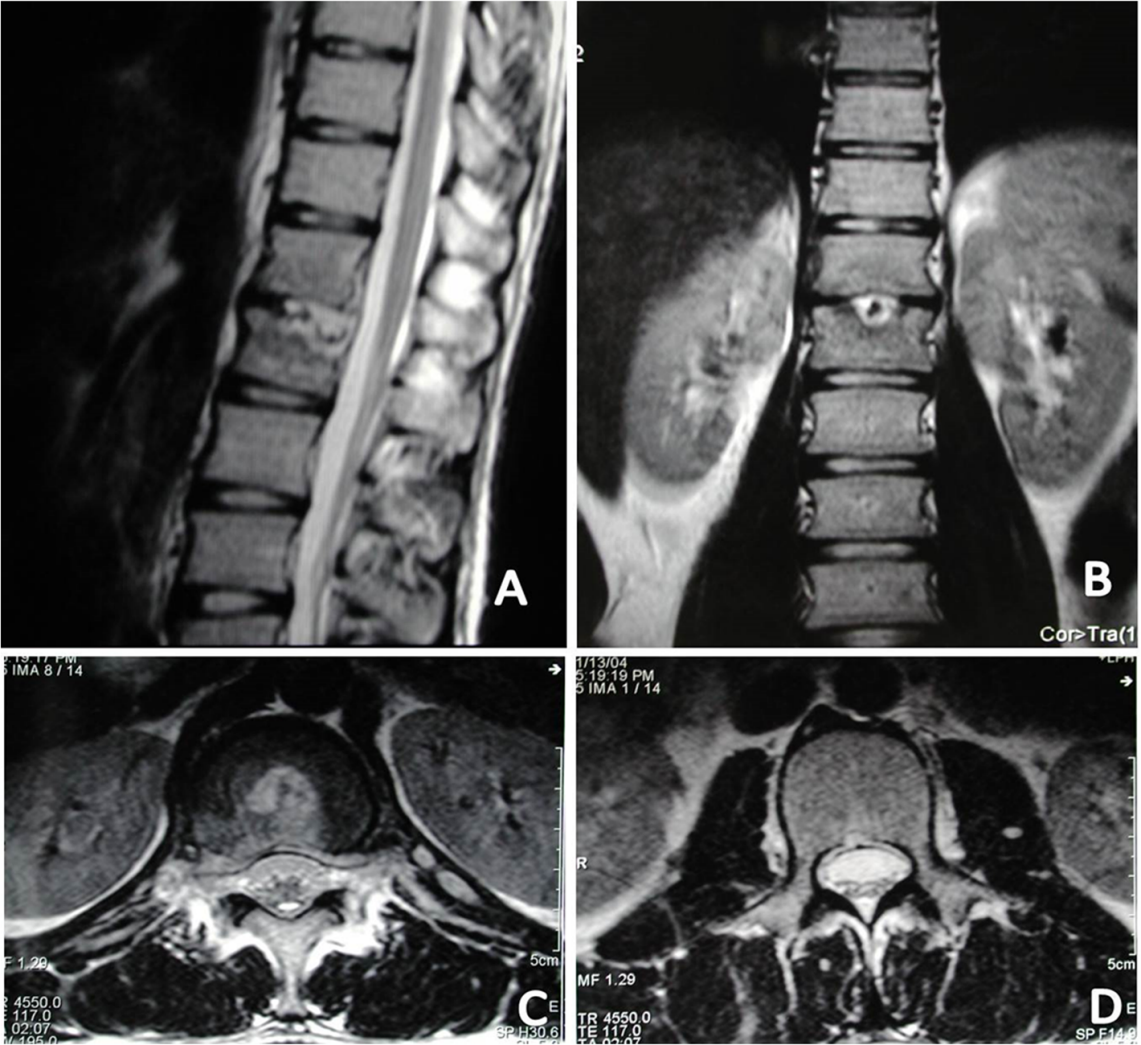
24 months after PCD and PCI chemotherapy, The MRI showed that intervertebral lesion were totally under control (**a**) with the right kidney was back to its right place and no abscess were observed in the bilateral psoas muscles in the coronal MRI (**b**) and in the axial MRI (**d**), and that the para-vertebral abscess also disappeared and the spinal cord were decompressed

ESR and CRP levels were dynamically monitored preoperatively and post-operatively at regular intervals to evaluate the infection status. The visual analog scale (VAS) was used to evaluate symptom relief and the Oswestry disability index (ODI) was used to assess rehabilitation condition and patient activity. Follow-up MRI was performed to assess the PA status. Treatment success was defined as short- and long-term absence of symptoms and improvement in laboratory and findings (Fig. 4 and Fig. 5). Total recovery was defined as absence of recurrent infection or abscess, bony fusion, and relief from back pain.

### Statistical analysis

SPSS 24.0 software (SPSS Inc., Chicago, IL) was used for the statistical analysis. The data were expressed as means ± standard deviations, and differences with a *P*-value < 0.05 were considered significant. The sample size was calculated using PASS 19.0.3 statistical software (NCSS Inc., USA). Sample size of cross-sectional study was calculated as below. Zα/2 is the parameter of significance testing(α = 0.05, Z0.05/2 = 1.96). P is the expected effective rate. According to the literatures reviewed [1, 3, 18], the effective rate is about 50% on the basis of preliminary estimation. Permissible error(δ) is in the range of 0–10%. To keep the accuracy of study, the Permissible error will be set as 10%. The design effect (deff) will be 1.5. According to the result of the calculation, the sample size of adequacy is 74. So the study is powered, since the sample size of the study is 106.

## Results

The most common symptom of ST was back pain, which was observed in all 106 patients preoperatively. The preoperative and postoperative ODI and VAS was recorded and analyzed (Table 2).

**Table 2:** VAS and ODI findings preoperatively and at 6 months

	Preoperative	Postoperative	***P***-value
**Back pain VAS**	6.5 ± 3.0	2.4 ± 2.3	0.006
**ODI**	33.6 ± 12.3	8.1 ± 4.6	0.011

*VAS* visual analog scale; *ODI* Oswestry disability index

**Fig. 6:**
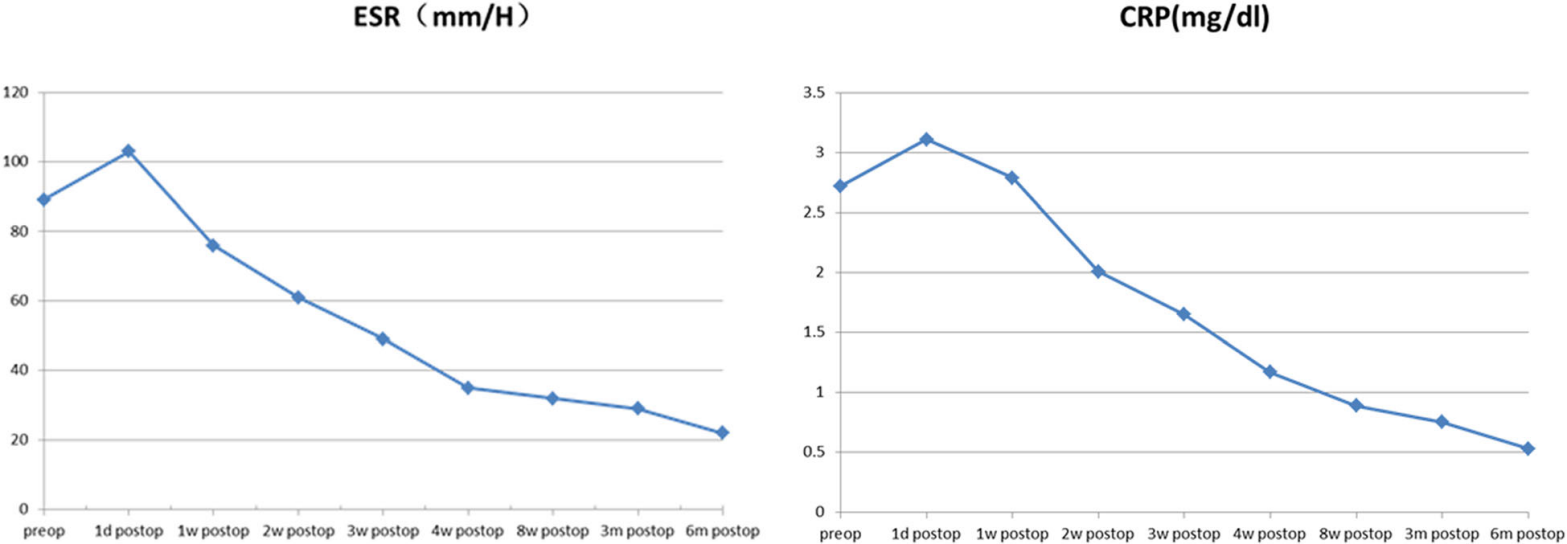
Statistical analysis demonstrated that there was significant difference between preoperative and eight weeks postoperative ESR and CRP (*P* < 0.05)

The elevated ESR and CRP levels returned to normal at a mean period of 3 months postoperatively. Statistical analysis demonstrated a significant difference in ESR and CRP levels preoperatively and at 8 weeks postoperatively (*P* < 0.05) (Fig. 6).

Four patients developed recurrent abscess during the follow-up period at 5, 9, 17, and 22 months, respectively. Among these 4 patients, 3 underwent repeated minimally invasive PCD and PCI and were subsequently cured. The remaining patient developed an iliac fossa abscess and underwent complete retroperitoneoscopic debridement [19].

During PCI chemotherapy, the infusion tubes of 14 patients were accidentally pulled out. The patients subsequently underwent re-placement and resumed chemotherapy.

The mean follow-up period was 7.21 ± 3.15 years. One patient died of pneumonia at 8 years postoperatively and another died of lung cancer at 11 years, with no recurrent infections or abscesses at the last follow-up examination in each. Seven patients underwent two-stage percutaneous pedicle screw fixation surgery to prevent severe kyphotic deformity (> 45°) after the MIS procedure [13]. At final follow-up, 97 patients were free from back pain and 7 had occasional back pain. Solid bony union was observed in 106 patients and no abscesses were found on MRI examinations. No other obvious complications were observed.

## Discussion

The WHO estimates that there are about 10.4 million new cases and 1.8 million deaths from TB each year. One-third of these new cases (approximately 3 million) remain unknown to healthcare systems, and many patients are not receiving proper treatment [20]. TB is an infectious bacterial disease caused by *Mycobacterium tuberculosis*, which is transmitted between humans through the respiratory route. While it most commonly affects the lungs, it can damage all tissues [18]. About 10% of all TB cases present with musculoskeletal involvement, of which 50% involves the spine. The thoracic and lumbar spine are the most commonly involved sites (90% of cases) [3].

The treatment regimens for thoracic and lumbar TB reported in the English literature include anti-TB chemotherapy (usually combined treatment with isoniazid, rifampicin, pyrazinamide, and ethambutol) [2], traditional surgical treatment (anterior radical debridement with graft fusion or posterior debridement with fusion and fixation) [21–25], and minimally invasive approaches [19, 26–32].

The literature supports anti-TB chemotherapy as the gold standard treatment for ST [2, 18, 33]. Additional surgical intervention is performed if there is evident vertebral instability, failure of chemotherapy, progressive deformity, serious neurological impairment, or presence of extensive PA with or without epidural involvement [22, 24, 25, 29–32].

As to vertebral instability and progressive deformity, percutaneous pedicle screw fixation should be performed to prevent vertebral collapse and kyphotic deformity. In the presence of extensive PA, PCD [34–41] or retroperitoneoscopic drainage [19, 27, 28] are the recommended treatments.

Open surgery is required to confirm debridement of the lesion or abscess and decompression of the spinal cord or nerve roots if patients exhibit serious neurological impairment [42]. According to Hodgson et al. [43], paraplegia associated with ST can be classified into two groups: neurological impairment for less than 2 years or for more than 2 years. Impairment of shorter duration is associated with active disease and compression caused by the abscess and inflamed tissue. Longer duration of impairment is related to vertebral collapse, spinal deformity, and secondary compression [3].

Numerous studies on new and individualized treatment regimens for ST have been conducted [44–48]. For patients with thoracic or lumbar TB and PA, but no serious neurological impairment or angular kyphotic deformity, the management strategy should be focused on minimally invasive debridement and drainage of the abscess in addition to chemotherapy [19, 28]. PCD for ST with PA has been shown to be safe and effective [34, 36, 38, 40, 45, 48, 49]. In our study, we focused on the outcomes of MIS involving percutaneous catheter drainage PCD and PCI chemotherapy. Local chemotherapy is critical to ensuring an adequate drug concentration reaches the tubercular lesion [18, 33, 50].

### The similarities and differences

The treatment regimens for thoracic and lumbar TB remained controversial. Individualized medical regimen need to be administrated according to the different situations of spinal tuberculosis. The suitable intervention method should be found out for the corresponding indications.

So the intervention regimens for thoracic and lumbar TB reported in the English literature include traditional open surgical treatment [25, 51] and minimally invasive approaches (percutaneous catheter drainage) [46, 48] on the basis of systematic anti-TB chemotherapy [21].

1. The similarities include alll treatment is administered on the basis of the systematic anti-TB chemotherapy [45].
2. The differences between our MIS method and traditional open surgical treatment were including indication and intervention method [24] as follow:

The indication for the MIS method including: (1) paravertebral abscess or posas abscess secondary to spinal tuberculosis involving the T8-L5 vertebrae, (2) without severe destruction of the vertebral body and obvious kyphosis or spinal instability, (3) without serious nerve or spinal cord compression symptoms. When the situations such as (2) or (3) occurs, open surgical operation is recommended instead of the MIS method [27, 28, 44].

The intervention method of the MIS method is percutaneous while the open surgery including anterior radical debridement with graft fusion or posterior debridement with fusion and fixation with huge invasiveness and more blood loss [22, 52].

The differences between our MIS method and the minimally invasive approach (percutaneous catheter drainage) were clarified below. Our MIS method involved PA elimination by percutaneous catheter drainage (PCD) and intervertebral percutaneous catheter infusion (PCI) chemotherapy for treating the TB lesions while percutaneous catheter drainage only focused on the drainage of abscess. Percutaneous catheter infusion (PCI) and irrigation with high concentrations of chemotherapy can eliminate remnant tubercle bacilli in the abscess cavity and reduce the rate of abscess recurrence [38, 41].

### The principals of MIS

Considering the enrollment criteria, this study focused on MIS for treatment of the abscess and tuberculous vertebrae [28]. While CT-guided percutaneous drainage for ST had already been reported [15, 24, 34, 53–55], its outcomes have not been clearly and definitively elucidated. In addition, no large-scale, long-term studies have been conducted to investigate the efficacy of a minimally invasive approach involving PCD and PCI chemotherapy. The diagnostic and therapeutic value of PCD with PCI chemotherapy, owing to complete abscess drainage and adequate drug delivery to the infected vertebrae, has been previously reported [56–58]. Since infection of the T8–L5 vertebrae is common, PA and psoas abscess are not rare entities [15, 34, 55]. In fact, PAs, which include subdural and perivertebral abscesses, are one of the most common complications observed in the T8–L5 region [6]. In contrast, the anatomical location of the psoas abscess muscle means that psoas abscesses usually occur in the context of T12–L5 infection [4].

### The advantages of PCD and PCI chemotherapy

The advantage of PCD is the continuous and complete drainage with postoperative negative-pressure Hemovac suction [57]. Furthermore, sticky abscesses can be washed out by pressurized irrigation with saline and isoniazid. Irrigation with high concentrations of isoniazid can eliminate remnant tubercle bacilli in the abscess cavity and reduce the rate of abscess recurrence [59].

The PCI approach enables precise and daily delivery of isoniazid to the tuberculous foci [60]. A high local drug concentration has been confirmed as the key factor for treatment of infection foci and avoiding recurrent infection [59, 61, 62]. Furthermore, debridement of the infected disc and adjacent vertebral endplates can be completed through the working tube during catheterization [24]. This minimally invasive technique leads to less morbidity than major open surgery, and provides effective relief of the patients’ back pain by reducing the intradiscal pressure and preserving spinal stability [13, 14, 28]. Therefore, our patients were able to start ambulating with brace protection as early as possible after PCD and PCI chemotherapy.

## Limitations

This study has several limitations. First, the retrospective nature of this study lacks random assignment of patients and does not allow for comparison of the outcomes of different treatment methods. Second, due to the absence of a control group, it is difficult to definitively state that the MIS technique is superior to open surgery. Third, the feasibility and benefits of PCD and PCI chemotherapy in patients with extended indications, such as postoperative recurrent infection or multilevel involvement, need to be rigorously evaluated in controlled, prospective studies with large patient populations.

## Conclusion

Based on the findings observed in this study, we propose that a minimally invasive approach involving PCD and PCI chemotherapy is an effective option for the treatment of thoracic and lumbar TB with PA and/or psoas abscess. Major open surgery is not always necessary in these cases.

## Data Availability

Date that support the findings of this study are available from the corresponding author on reasonable request.

## Abbreviations

MIS: Minimally invasive surgery
PA: Psoas abscess
ST: Spinal tuberculosis
TB: Tuberculosis
PCD: Percutaneous catheter drainage
PCI: Percutaneous catheter infusion
CRP: C-reactive protein
ESR: Erythrocyte sedimentation rate
PPD: Purified protein derivative
IGRA: Interferon gamma release assay
CT: Computed tomography
MRI: Magnetic resonance imaging
VAS: Visual analog scale
ODI: Oswestry disability index
